# Supplementary material for: Immunomodulatory role of Keratin 76 in oral and gastric cancer
Source: Nat Commun. 2018 Aug 24;9:3437. doi: 10.1038/s41467-018-05872-4 (PMC6109110; doi:10.1038/s41467-018-05872-4)
Supplement: Supplementary file 1 — Supplementary Information [file 41467_2018_5872_MOESM1_ESM.pdf]

Supplementary Information

Immunomodulatory Role of Keratin 76 in Oral and Gastric Cancer

Sequeira et al.

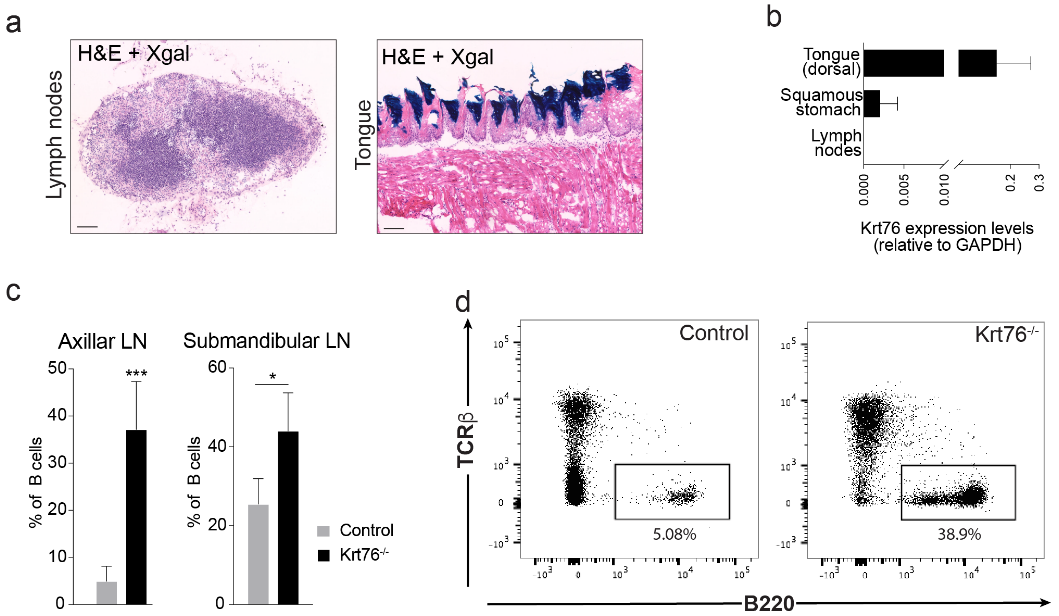

Supplementary Figure 1 – Krt76 is not expressed in the lymph nodes but loss of Krt76 results in expansion of B cells in lymph nodes. (a) Representative images of Xgal staining of control Krt76$^{+/-}$ mouse lymph node, showing lack of Krt76 expression, and tongue expressing Krt76 as a control (n=3 mice). Scale Bars = 100μm. **(b)** Krt76 mRNA qRT-PCR analysis of adult tissues, relative to Gapdh (n=3 mice, experiment repeated twice, means ± s.e.m. are shown). **(c)** Summary of flow cytometric analysis of % B cells (TCRβ^{-} B220^{+}) in total cells from lymph nodes of control and Krt76$^{-/-}$ mice (n=4 mice/genotype, means ± s.e.m., *p≤0.05, ***p≤0.001, unpaired t-test). **(d)** Flow cytometry plots of B cells (TCRβ^{-} B220^{+}). Plots are representative of 2 experiments, n=4 mice/genotype/experiment.

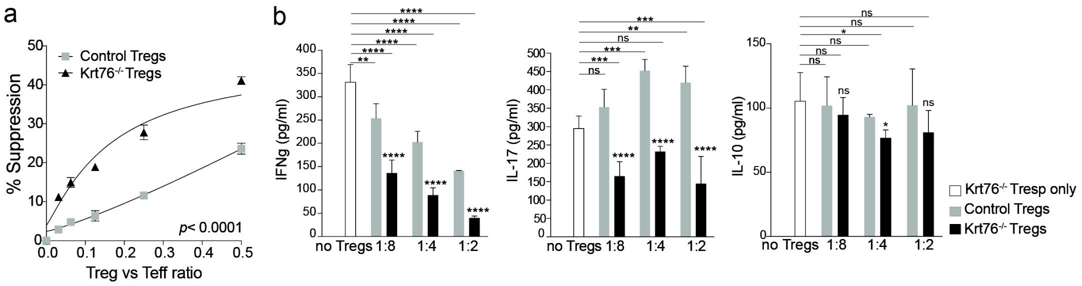

Supplementary Figure 2 – Suppressive function of Krt76$^{-/-}$ Tregs in the presence of Krt76$^{-/-}$ Tresp.

(a) Cumulative data showing % Krt76$^{-/-}$ Tresp suppression at each Tresp:Treg ratio in the presence of control or Krt76$^{-/-}$ Tregs (mean ± s.e.m, 2 independent experiments, 2-way ANOVA, $p<0.0001$, Non-linear regression (curve fit) of Treg suppression). **(b)** Levels of cytokines in cell culture medium at each Tresp:Treg ratio, assessed by CBA analysis (means ± s.e.m., multiple t-tests and two-way ANOVA, each culture condition in triplicate, measured in duplicate, experiment repeated twice). *$p\leq0.05$; ***$p\leq0.001$; ****$p\leq0.0001$; ns, non-significant.

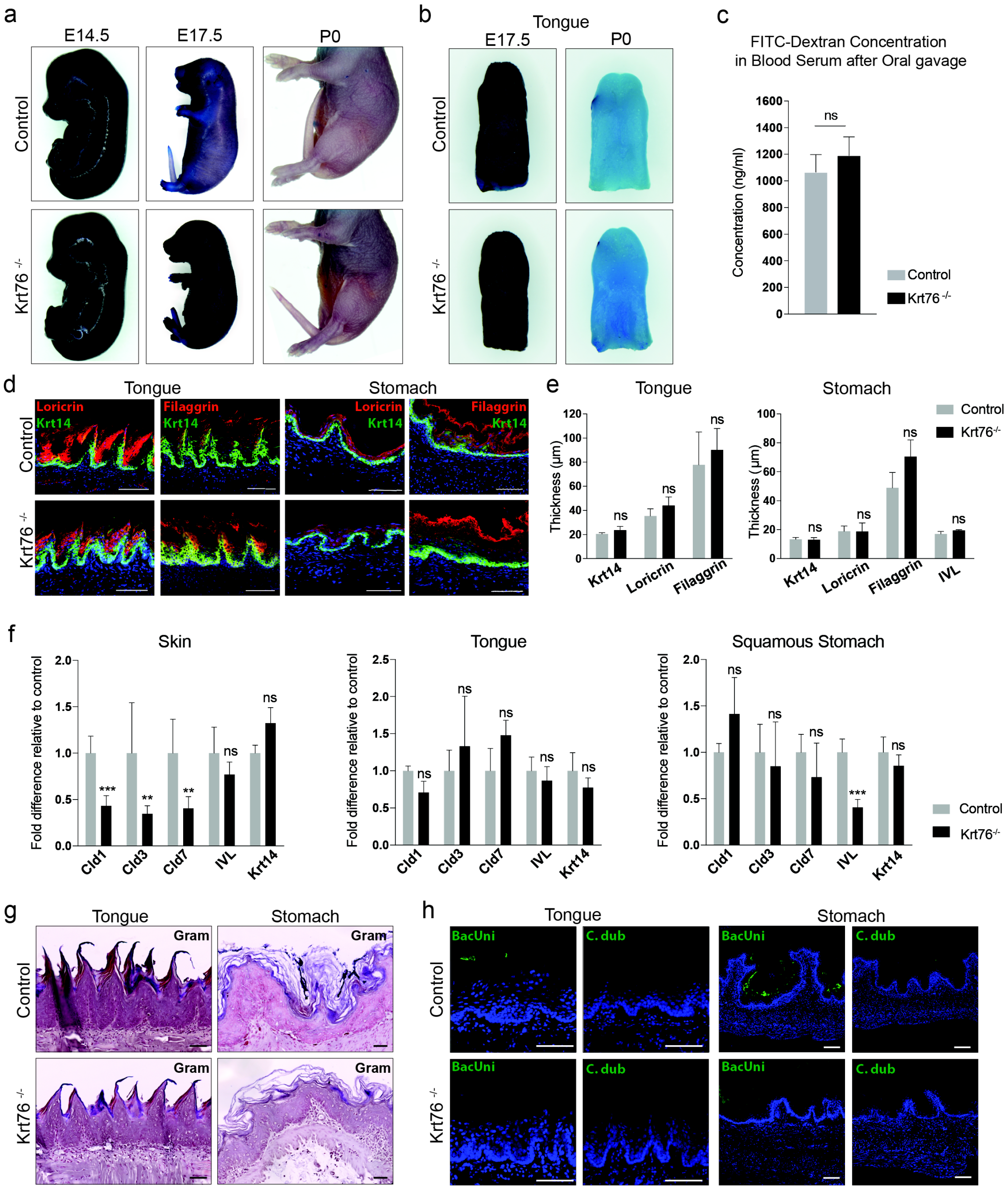

Supplementary Figure 3 – Loss of Keratin 76 does not alter the epithelial integrity or permeability of tongue and stomach.

(a, b) Toluidine blue permeability assay of control and Krt76$^{-/-}$ mouse embryos and dissected tongues at E14.5, E17.5 and P0. **(c)** Stomach permeability measured by determining the concentration of FITC-dextran in the serum of control and Krt76$^{-/-}$ mice (n=4 mice/group, means ± s.e.m; p=0.178; unpaired t-test). **(d)** Immunofluorescence labelling of differentiation markers Loricrin and Filaggrin (red) and Krt14 (green) in tongue and stomach epithelia of adult mice. **(e)** Quantification of thickness of each labelled layer using ICY software (n=3 mice per genotype, means ± s.e.m., unpaired t-test). **(f)** mRNA qRT-PCR analysis of tight junction and differentiation markers in tongue and stomach epithelia of adult mice (n=4 mice per genotype, experiment repeated twice, means ± s.e.m., unpaired t-test). **(g)** Representative images of Gram-staining in tongue and stomach epithelia of adult mice. **(h)** Epithelial penetration by bacteria analysed by FISH using BacUni or *C. dubuliniensis* (control) probes (green), with nuclear DAPI counterstain (blue). The images are Z-stack maximum projections of tongue and stomach epithelia. **p≤0.01; ***p≤0.001; ns, non-significant. Scale Bars = 200µm (d, g, h).

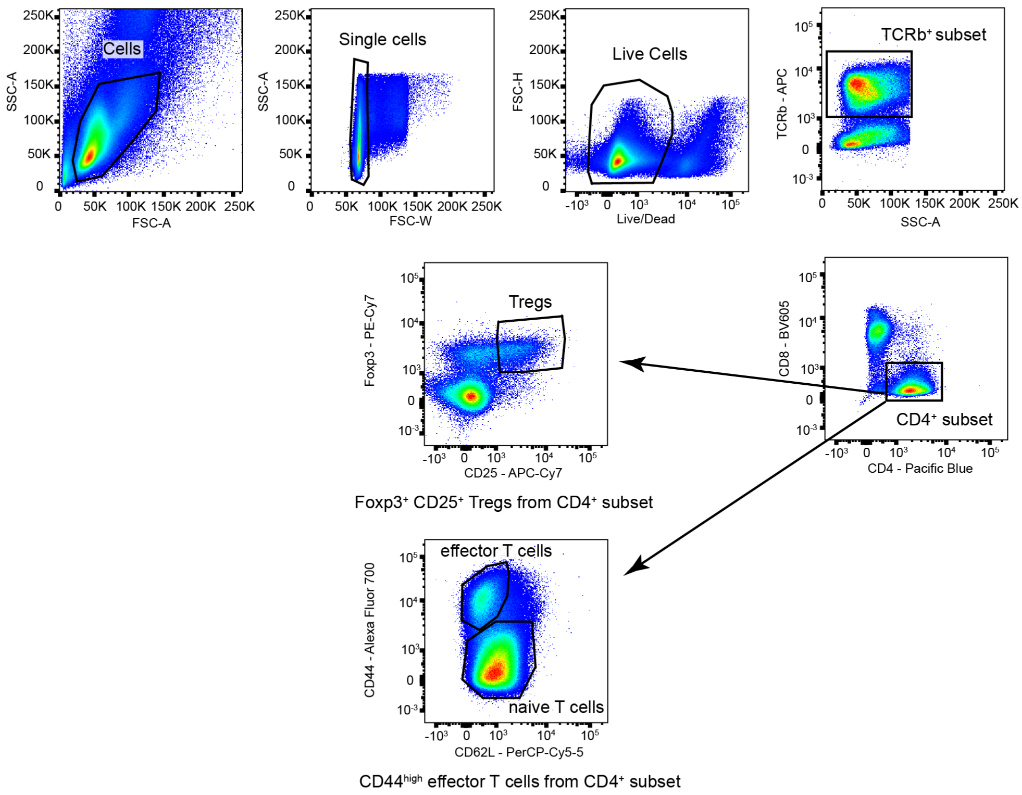

Supplementary Figure 4 – Flow cytometry gating strategy. Representative plots showing gating strategy used to isolate Foxp3+ Tregs and effector T cells, initially gating on forward and side scatter profiles (left panel), then identifying live cells, selecting CD4+ T cells, and selecting CD25+ Foxp3+ Tregs or CD44^{high} CD62L^{low} effector T cells within the CD4+ T cell population.

Gene name	Probe ID (in ThermoFisher Scientific)
Krt76	Mm01305200_m1
IGNγ	Mm01168134_m1
IL-2	Mm00434256_m1
IL-4	Mm00445259_m1
IL-6	Mm00446190_m1
IL-10	Mm01288386_m1
TNFα	Mm00443258_m1
IL-1a	Mm00439620_m1
TSLP	Mm01157588_m1
HMGB1	Mm00849805_gH
GAPDH	Mm99999915_g1

Supplementary Table 1 – List of TaqMan probes.
